# Serological and Pathogenic Analyses of Fowl Adenovirus Serotype 4 (FAdV-4) Strain in Muscovy Ducks

**DOI:** 10.3389/fmicb.2018.01163

**Published:** 2018-06-01

**Authors:** Xianglong Yu, Zhenzhong Wang, Hao Chen, Xiaoyu Niu, Yanguo Dou, Jing Yang, Yi Tang, Youxiang Diao

**Affiliations:** ^1^College of Animal Science and Technology, Shandong Agricultural University, Tai’an, China; ^2^Shandong Provincial Key Laboratory of Animal Biotechnology and Disease Control and Prevention, Tai’an, China; ^3^Shandong Provincial Engineering Technology Research Center of Animal Disease Control and Prevention, Tai’an, China

**Keywords:** clinical investigations, experimental infection, fowl adenovirus serotype 4, muscovy ducks, serological test

## Abstract

Hydropericardium hepatitis syndrome (HHS) is a lethal disease caused by Fowl adenovirus serotype 4(FAdV-4) that mainly infects 3- to 6-week-old broiler chicks. In 2015, an infectious disease characterized similar symptom to HHS in broilers outbroke in commercial duck flocks in Shandong province. FAdV-4 was isolated from naturally infected ducks and determined by polymerase chain reaction (PCR) amplification and DNA sequence analysis. In order to investigate the effect of FAdV-4 infection on muscovy ducks, we determined and characterized the FAdV-4 Isolate, and assessed its pathogenicity. In this study, HHS was respectively reproduced in 5-week-old muscovy duck by intramuscular injection and intranasal inoculation of allantoic fluid containing FAdV-4, ducks in the negative control group were inoculated with allantoic fluids of healthy duck embryos in the same manner. Clinical symptoms, gross and microscopic lesions, cytokines and antibodies, blood biochemical indices were detected and recorded for 12 days after infection. Typical hydropericardium and hepatitis was observed in experimental muscovy duck in the 3rd day post-inoculation (dpi). FAdV-4 can be replicated in tissues and cause pathological damage, especially in the liver and immune organs. Most of the immune-related cytokines and antibodies levels are up-regulated and then decreased, which may be caused by the initial infection and the normal immune response, later the virus caused the immunosuppression and led to the decrease of levels. To the best of our knowledge, this is the first systematic trial of the pathogenicity of FAdV-4 in muscovy ducks mainly based on the serological test, which will provide new insights into the study of the disease.

## Introduction

Fowl adenoviruses (FAdVs) is non-enveloped double stranded DNA-viruses, which was grouped into 5 species (FAdV-A to FAdV-E) with 12 serotypes (FAdV-1 to 12) (Hess, 2000). Hydropericardium hepatitis syndrome (HHS) was first reported in Angara Goth, Pakistan, which characterized by accumulation of colorless or slightly yellow transparent fluid in the pericardial sac and hepatitis with characteristic intranuclear inclusion bodies in hepatocytes, with a high mortality of 30–70%. Outbreaks of HHS caused by FAdV-4, and the disease have occurred all over the world (Mase et al., 2010; Schachner et al., 2018).

Previous study indicated that FAdV-4 isolates can cause HHS and significant mortality in broilers and goose (Toro et al., 2000; Ivanics et al., 2010). In 2015, an infectious disease, which mainly infected 25–40 day-old ducklings, characterized similar symptom to HHS, outbroke in commercial duck flocks in Shandong province. According to lately published report, this disease was confirmed to be caused by FAdV-4 (Chen et al., 2017). In this study, one FAdV-4 strain which isolated from recent outbreaks was characterized by phylogenetic assays and pathogenic analysis. Briefly, 4-week-old ducks were infected with FAdV-4 by different routes to investigate the effect of FAdV-4 on muscovy ducks.

## Materials and Methods

### Ethics Statement

All applicable international, national, and institutional guidelines for the care and use of animals were followed to minimize suffering. Animal procedures was approved by the Committee on the Ethics of Animal of Shandong (permit number: 2017360331). Thirty-six 4-week-old muscovy ducks were used in the experiment and maintained at Shandong Agricultural University with water and food *ad libitum*, relative humidity 40–60% conditions and a 12–12 h light-dark cycle. Ducks were euthanized using rapid cervical dislocation.

### Animals

Four-week-old muscovy ducks were purchased from Wen’s Group Company which is under controlled sanitary status in Fujian. *Ad libitum* feeding and drinking were provided for ducks in specific pathogen free (SPF) chicken isolators. Serum and swab samples were collected from ducks prior to inoculation and detected to confirm that all ducks used in this study were serologically negative for FAdV-4 by serum neutralization test and PCR test.

### Clinical Investigations

We investigated about 500 ducks showing HHS-like symptoms and collected 112 liver and lung samples from the sick and dead ducks in several farms in Shandong province from July 2013 to January 2015. The sites include Taian, Liaocheng, Shenxian, Juxian and Linyi. Samples were collected and kept at -4°C until being detected.

### Virus Isolation and Identification

All livers collected from HHS-suspected ducks were homogenized with normal saline and filtered through 0.22 um filters. The virus was isolated by LMH cells (Cell Type: chemically induced) and chorioallantoic membrane (CAM) route following standard method (Samuel et al., 1981). The virus was passaged five times via CAM route and the titer was determined to be 10^-7.60^ EID_50_/0.2 ml by Reed and Muench method. Five samples of allantoic fluids from different farms were used for sequencing. Allantoic fluids were also harvested as the challenge virus in the present study and stored at -80°C.

### DNA Sequencing

DNA was extracted by phenol–chloroform method from liver samples which presented with enlargement and hemorrhage (Kochl et al., 2005). To detect the virus, the PCR assay was performed with a pair of self-designed primers, and the design of primers were based on the published sequence strain SDSX1 (GenBank Accession No. KU845700.1) of the hexon gene (forward: TGGACATGGGGGCGACCTA; reverse: AAGGGATTGACGTTGTCCA). Primers specifically amplify a 1204-bp fragment of FAdV-4. Reactions were performed according to the following protocol: 95°C for 5 min, followed by 30 cycles of 95°C for 30 s, 53°C for 30 s, 72°C for 60 s, and a final elongation step of 10 min at 72°C. PCR products were analyzed by agarose gel electrophoresis.

All positive PCR products were cloned into pMD18-T vector, transformed into DH5α *E. coli* competent cells and sequenced using the Sanger dideoxy sequencing method (BGI Company Ltd., Beijing, China). Complete sequences were aligned with other available FAdV genome sequences to determine the nucleotide sequence homologies using the MegAlign program of the DNAstar software suite (version 8.13, DNAstar, Madison, WI, United States). Phylogenetic analysis which based on the nucleotide sequence of hexon gene of the positive samples and other FAdV was constructed using MEGA7 software with neighbor-joining method.

### Experimental Procedure

To determine the pathogenicity of FAdV-4, 4-week-old ducks (12 control ducks and 24 inoculated ducks, respectively) were randomly divided into three different groups (two groups as experimental groups and another group as the negative control group) and housed in separate isolators. Ducks in experimental groups were inoculated by intranasal route and intramuscular route, respectively with 0.3 mL 10^-7.60^ EID50/0.2 ml of the challenge virus and ducks in the negative control group were inoculated with allantoic fluids of healthy duck embryos in the same manner. Ducks were continuously observed for 12 days, and clinical symptoms were recorded after infection. On 1, 3, 6, 9, and 12 days post-inoculation, three ducks from each group were euthanized and their tissues (heart, liver, spleen, lung, kidney, thymus, and bursa of Fabricius) were collected and fixed with 4% paraformaldehyde solution. About 1 week after, all fixed tissues were embedded paraffin, sectioned, and stained with hematoxylin and eosin for histopathological examination. Moreover, portion of tissues per group were collected and stored at -80°C for DNA extraction according to phenol-chloroform method and the PCR test was performed with a pair of specific primers (forward: TGGACATGGGGGCGACCTA; reverse: AAGGGATTGACGTTGTCCA) according to the Chen’s method (Chen et al., 2017). Serum samples were collected from all ducks before sacrifice and stored at -20°C until using for detecting and recording the changes of antibodies, cytokines and blood biochemical indices. Cytokines were detected by using the sandwich ELISA Kit of duck IL-2, IL-4, IL-6, and IFN-γ (Langton, Shanghai, China) According to the manufacturer’s directions. Immunoglobulins were also detected by using the sandwich ELISA Kit of duck IgG, IgM and IgA (Langton, Shanghai, China). The FAdV-4-specific antibodies were determined by the indirect ELISA which developed in previous work in our laboratory. For Indirect ELISA, 47 μg/mL recombinant hexon protein in carbonate buffer solution was incubated in black Maxisorb 96-well plates overnight at 4°C and the plates were blocked by 5% non-fat dry milk for 2 h at 37°C, then serum (diluted to 1:10 with non-fat dry milk) was added at 100 μL/well. Biochemical indices, including alanine aminotransferase (ALT), aspartic aminotransferase (AST), lactic dehydrogenase (LDH) and alkaline phosphatase (ALP), were detected through a testing organization called ADICON CLINICAL LABORATORIES, INC. (Jinan, China).

### Statistical Analysis

All data were expressed as means ± standard deviation (SD) and analyzed using the One-Way Anova procedure of GraphPad Prism 6.0 (GraphPad Software Inc., San Diego, CA, United States). Statistical significance was set at *P* < 0.05 or *P* < 0.01.

## Results

### Results of Investigation and Virus Identification

**FIGURE 1:**
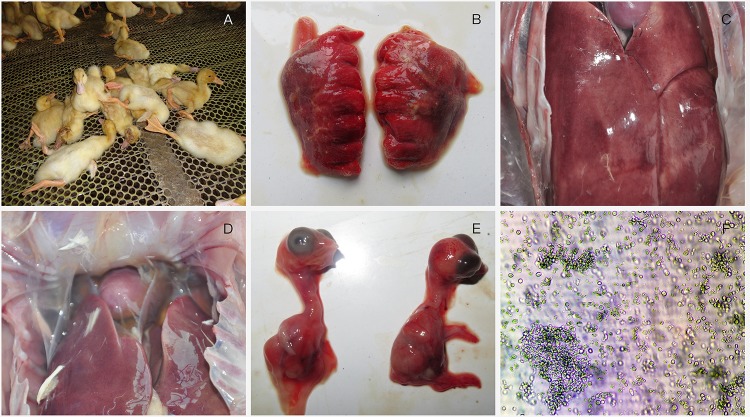
Clinical investigation and virus identification. **(A)** a large number of ducks died suddenly; **(B)** lungs showing hemorrhage; **(C)** Liver is enlarged with blood spots; **(D)** pericardial effusion; **(E)** 13-day-old chicken embryos infected with FAdV-4 were dead with stunted growth and severe cutaneous hemorrhages at 96 h post-inoculation; **(F)** Severe cytopathic effects were observed in LMH cells.

In HHS-contaminated farms, no significant clinical signs but the sudden death, with the mortality of 10–30%, can be observed in infected ducks (**Figure 1A**). Post-mortem examination revealed severe gross lesions in most ducks, including hemorrhage of the lungs, hemorrhaging and enlargement of livers and pericardial effusion in heart (**Figures 1B–D**).

Chicken embryos died at 3–5 dpi, with stunted growth and severe cutaneous hemorrhages compared with healthy embryos (**Figure 1E**). Severe cytopathic effects (CPE) were also observed in LMH cells including necrosis and separate plaque formation (**Figure 1F**).

### Sequence Analysis

**FIGURE 2:**
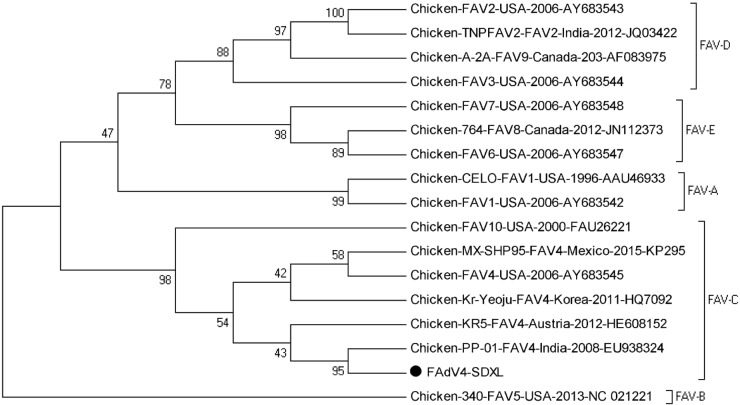
Phylogenetic tree of hexon gene sequences of the isolate SDXL and other 16 fowl adenovirus strains. The tree was constructed by the neighbor-joining method with 1000-bp replicates using MEGA 7.0 software.

Ninety-two positive samples were sequenced and the strain designated SDXL (GenBank accession: MG209111) was isolated from liver samples. Multiple alignments showed that the isolate shared hexon sequence identities of 97.9–99.3% with other FAdV-4 isolates (Kr-Yeoju, KR5 and PP-01) and the deducted amino acid sequences of hexon genes of isolate SDXL shared 96.7–98.8% identities with those of reference isolates. As shown in **Figure 2**, the phylogenetic tree for hexon gene revealed that the isolate SDXL was clustered together with chicken FAdV-4 isolates (Kr-Yeoju, KR5 and PP-01) (Steer et al., 2009).

### Clinical Symptoms and Gross Lesions

The ducks of the control group did not show any clinical symptoms or necrotic lesions throughout the experiment. Two ducks of experimental groups showed purplish red diarrhea at 6 dpi and three ducks showed yellow water-like diarrhea at 9 dpi. No death occurred from post-inoculation to the end of the experiment.

**FIGURE 3:**
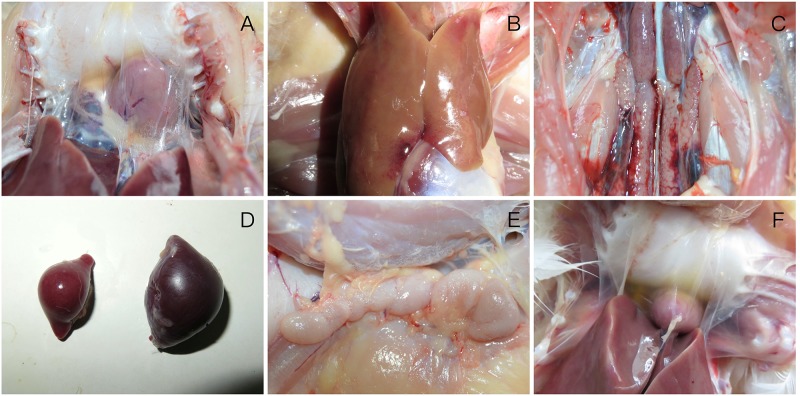
Gross lesions of FAdV-4 -infected ducks **(A)** mild pericardial effusion; **(B)** swollen livers with bleeding spots; **(C)** kidneys with hemorrhaging and enlargement; **(D)** enlargement of spleen; **(E)** enlargement of thymus; **(F)** severe pericardial effusion in heart.

At necropsy, the mild pericardial effusion of one intramuscularly inoculated duck was a significant lesion at 3 dpi (**Figure 3A**), and they also demonstrated swollen livers with bleeding spots (**Figure 3B**), kidneys with hemorrhaging and enlargement (**Figure 3C**). At 6 dpi, each euthanized duck presented significant lesions characterized by haemorrhagic enteritis in the intestine, earth yellow and soft livers, bleeding of thymus and enlargement of spleen (**Figure 3D**) and serious pericardial effusion. At 9 dpi, bursa of Fabricius and thymus was enlarged obviously (**Figure 3E**), and each organ lesions were more evident (**Figure 3F**). At 12 dpi, gross lesions showed a less severe trend.

Additionally, FAdV-4 was extracted from tissues (heart, liver, spleen, and lung) and confirmed by PCR test. The virus was also re-isolated by inoculating the SPF chicken embryos.

### Histopathologic Analysis

Histopathological analysis showed that both experimental groups ducks inoculated with FAdV-4 had obvious histopathological changes. The main microscopic lesions were observed in lung, kidney and liver.

**FIGURE 4:**
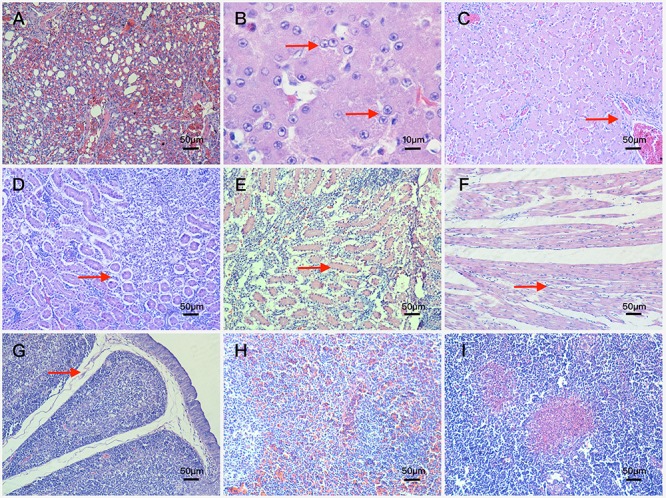
Histopathologic changes of FAdV-4-infected ducks **(A)** blood stasis and dilation in lung capillaries and terminal bronchioles. Magnification, × 200; **(B)** basophilic inclusion bodies in hepatocyte nucleus. Magnification, × 1000; **(C)** fatty degeneration of hepatocytes and severe lymphocyte infiltration around vessels. Magnification, × 200; **(D)** renal tubular epithelial cells falling away from glomerular basement membrane. Magnification, × 200; **(E)** swelling and necrosis of cells became severe in the kidney. Magnification, × 200; **(F)** interstitial edema and lymphocyte infiltration around cardiocytes. Magnification, × 200; **(G)** interstitial edema and obvious decrease in the number of lymphocytes in the bursa of Fabricius. Magnification, × 200; **(H)** amyloidosis and extensive congestion in interstitium in the spleen. Magnification, × 200; **(I)** focal necrosis in the thymus. Magnification, × 200.

In the lung, bronchioles structures were obliterated, meanwhile arterial hyperemia appeared in two muscovy ducks euthanized at 3 dpi. Blood stasis and dilation were observed in lung capillaries and terminal bronchioles at 6 dpi (**Figure 4A**).

In the liver, vessel walls (arteries and veins) were thickened mildly with lymphocyte infiltration and fibrinous inflammation at 3 dpi, besides these basophilic inclusion bodies in hepatocyte nucleus began to appear (**Figure 4B**). At 6 dpi, lesions in liver were characterized by interstitial hemorrhage accompanied with lymphocyte infiltration, venous congestion and the number of inclusion bodies increased significantly. At 9 dpi, the interstitial edema and severe interstitial hemorrhage were more frequently observed compared with the 6 dpi ducks, and further lesions including fatty degeneration of hepatocytes and severe lymphocyte infiltration around vessels were also observed (**Figure 4C**). At 12 dpi, the lesions of interstitial edema became mild, however, vessel walls were thickened more severely and inclusion bodies were more obvious in hepatocyte nucleus.

In the kidney, part of renal tubular epithelial cells fell away from their basement membrane at 3 dpi (**Figure 4D**), and swelling, degeneration and necrosis of tubular epithelial cells appeared at 6 dpi. At 9 dpi, most of tubular epithelial cells fell away from the basement membrane and part of the renal tubular structure disintegrated. Moreover, swelling and necrosis of cells became severe (**Figure 4E**).

Besides the above microscopic lesions, analysis also showed in the following aspects: Lesions in the heart were characterized by interstitial edema and lymphocyte infiltration around cardiocytes (**Figure 4F**); lesions in the bursa of Fabricius were characterized by interstitial edema and obvious decrease in the number of lymphocytes (**Figure 4G**); extensive congestion in interstitium was observed in the spleen (**Figure 4H**) and focal necrosis in the thymus (**Figure 4I**).

### Detection of Antibodies in Serum

**FIGURE 5:**
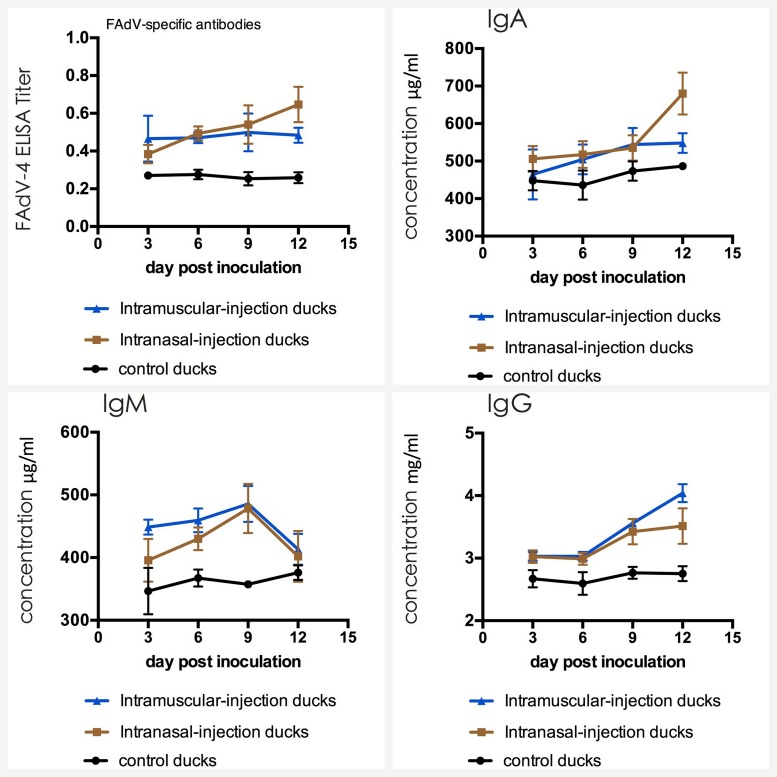
Dynamics of antibodies against FAdV-4 and IgA, IgM, IgG in serum of the ducks post artificially infected with FAdV-4.

The serum from each muscovy duck was collected for the detection of antibodies against FAdV-4 by indirect ELISA and immunoglobulins including IgG, IgM, and IgA by sandwich ELISA. The result of three groups was shown in **Figure 5** and no seropositive ducks in control group were observed. As shown in the line chart, positive serum could be detected earlier in intramuscular injection group at 3 dpi, compared with the intranasal injection group at 6 dpi. But the antibody titers of intranasal injection group continued to rise until exceed intramuscular injection group and reaching a maximum value at 12 dpi, which had a statistically significant difference compared with intramuscular injection group (*P* < 0.05).

The mean concentrations of IgA, IgM, and IgG in ducks of control group varied in a stable range (from 447 to 487 μg/ml for IgA, from 351 to 386 μg/ml for IgM and from 2.75 to 2.93 mg/ml for IgG). The average value of IgM in intramuscular injection group and intranasal injection group primarily increased to 455 μg/ml and 397 μg/ml at 3 dpi and the highest value was appeared at 476 μg/ml and 473 μg/ml at 9 dpi. Furthermore, the peak was very significantly higher than that of the ducks in negative control group (*P* < 0.01). At 12 dpi, the levels of IgA and IgG rapidly increased and these started to take over IgM which declined with time after 9 dpi (*p* < 0.01).

### Detection of Cytokines in Serum

**FIGURE 6:**
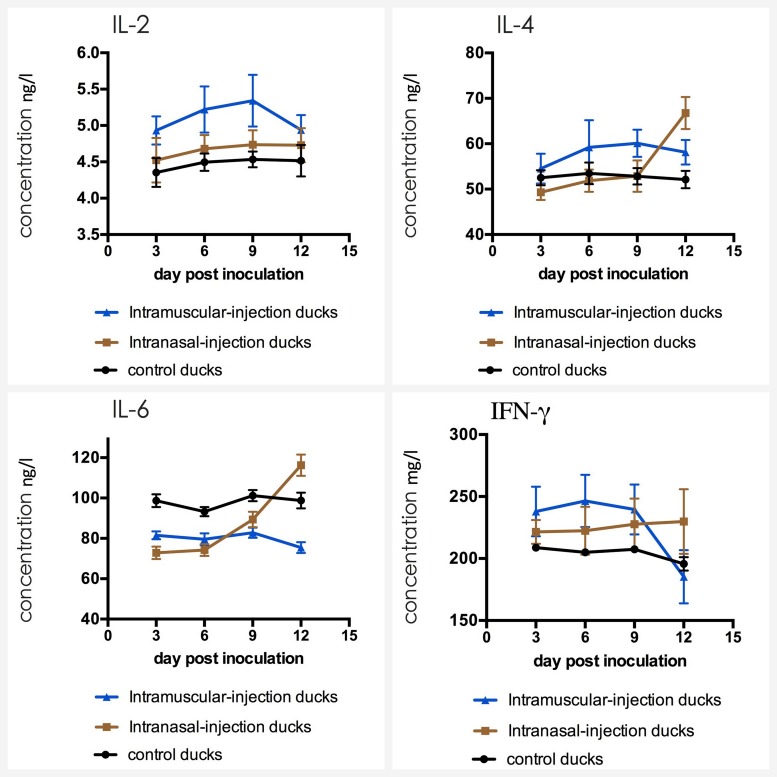
Dynamics of IL-2, IL-4, IL-6, and IFN-γ in serum of the ducks post artificially infected with FAdV-4.

The serum was carried out by sandwich ELISA to detect the level of cytokines. The result of three groups was shown in **Figure 6**. The mean concentrations of IL-2, IL-4, IL-6, and IFN-γ in ducks of control group varied in a stable range (from 4.39 to 4.57 ng/ml for IL-2, from 50.6 to 53.6 ng/ml for IL-4, from 91.7 to 99.6 ng/ml for IL-6 and from 196 to 209 mg/ml for IgG). IL-2 and IFN-γ levels of intramuscular injection group were up-regulated firstly, which had a statistically significant difference compared with that of control group when their levels reached the peak (*p* < 0.01). Then levels were down-regulated latter, and the level of IFN-γ even fell below that of the control group at 12 dpi. For IL-4, IL-6, the difference between intranasal injection group and control group was not obvious in the first 9 dpi (*p* > 0.05) meanwhile IL-4 level in intramuscular injection group was up-regulated slightly and IL-6 level was down-regulated slightly, but levels increased rapidly in intranasal injection group ducks from 9 dpi, which had a statistically significant difference compared with that of intramuscular injection group and control group (*P* < 0.05).

### Detection of Biochemical Indices

**FIGURE 7:**
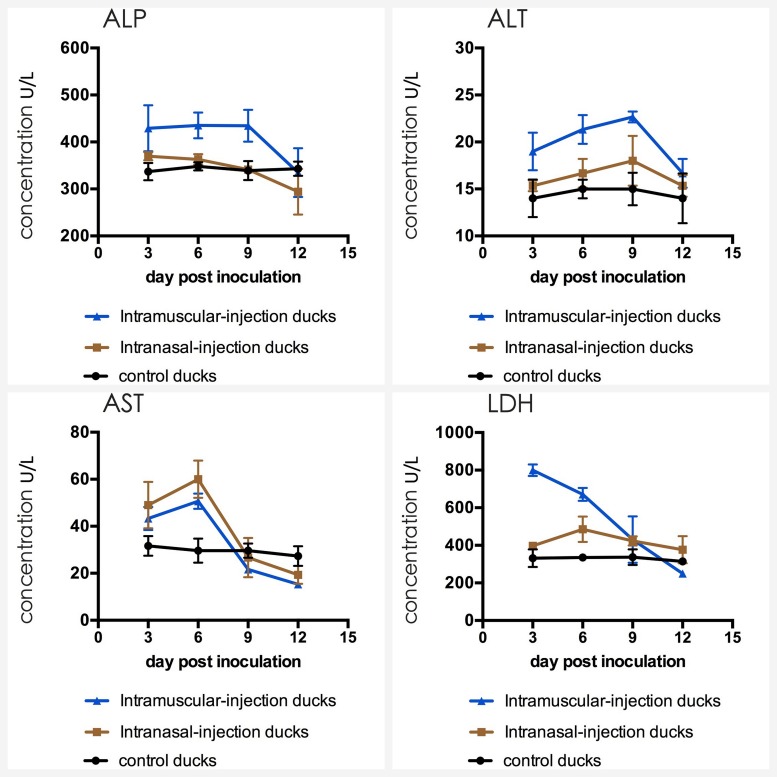
Dynamics of ALP, ALT, AST, and LDH in serum of the ducks post artificially infected with FAdV-4.

The detection result of three groups was presented in **Figure 7**. The analysis suggested that the AST, ALT and LDH of experimental groups were significantly higher than that of control group. Briefly, AST reached the peak at 6 dpi (*p* < 0.01), ALT at 9 dpi (*p* < 0.01), LDH of intramuscular injection group at 3 dpi (*p* < 0.01) and LDH of intranasal injection group at 6 dpi (*p* < 0.01). After peak levels, above biochemical indices declined with time. The ALP of intramuscular injection group was also up-regulated at first 9 dpi (*p* < 0.01) and down-regulated latter, but that of intranasal injection group were similar to the control group (*P* > 0.05).

## Discussion

Hydropericardium hepatitis syndrome caused by FAdV-4 was first detected and isolated from broilers in Eastern China during 2012 to 2013 (Zhao et al., 2015) and this disease was first detected from Cherry Valley ducks at 2015 (Chen et al., 2017). Currently, HHS has resulted in enormous economic loss for poultry industry (Asthana et al., 2013). As we know, Cherry Valley ducks and muscovy ducks are close relatives, and cultivation industry of muscovy ducks widely existed in South China. However, there was no report for muscovy ducks which can be infected by FAdV-4 and no study has yet to research the pathogenicity of FAdV-4 in muscovy duck. So, in this study, a systematic trial of the pathogenicity of FAdV-4 in muscovy ducks was carried out.

The outcome of FAdV-4 infection in muscovy ducks was partly related to inoculation method. Ducks of intramuscular injection group were more susceptible and severe to FAdV-4 infection compared to ducks of intranasal injection group. Most muscovy ducks of two experimental groups did not show obvious clinical symptoms or death during the tested period, but gross lesions observed in infected ducks, including pericardial effusion, swollen and yellowish livers with bleeding spot and bleeding or enlargement of immune organs (Kataria et al., 2013). Histopathological analysis also indicated obvious histopathological changes, including the interstitial edema and lymphocyte infiltration around cardiocytes, interstitial edema with hemorrhage and fatty degeneration in the liver, extensive congestion in interstitium in the spleen and focal necrosis in the thymus. Furthermore, gross lesions and microscopic lesions were largely consistent with the other latest reports (Chen et al., 2017).

Alanine aminotransferase and aspartic aminotransferase exist in hepatocytes in high concentration and the latter is also mainly present in cardiomyocytes (Fan et al., 2014). If hepatic cells or myocardial cells are destroyed, above enzymes will leak into the blood and levels of these will increase rapidly (Kew, 2000; Fan et al., 2003). LDH exists in the cytoplasm of all histiocytes in the body, and the content of LDH is highest in kidney. Therefore, LDH level can reflect tissue damages, especially for kidney’s damage. ALP mainly produces in hepatobiliary system, and hepatocytes will overproduce ALP if the hepatobiliary function is abnormal. The detection results of this study showed that almost all blood biochemical indices of experimental groups were higher than those of control group and then decreased with the time, which indicated that organs of the heart, liver and kidney were invaded after challenge and then cells may repair damages in themselves, and these were highly consistent with the Asrani’s report (Asrani et al., 1997). Furthermore, the changes of gross and histopathologic lesions also support the results of biochemical indices

IFN-γ is produced by thymus dependent lymphocytes (T cells) and natural killer cells (NK cells) and it is considered an essential cytokine for host defense against pathogens. IL-2 can promote proliferation and differentiation of T cells, and support the growth of T helper cells. In particular, the IL-2 content will decrease when the body is in an immunosuppressive state. In this study, the levels of IFN-γ and IL-2 in intramuscular injection group were up-regulated distinctly and were maintained in first 9 dpi, which reflected that T cells and macrophages produced a lot of these cytokines with high concentrations under the stimulation of FAdV-4. For the decrease from 9 dpi, the cause might be the destruction of the immune organs resulted from the virus replication or the immaturity of the immune system.

IL-4 and IL-6, also known as B cell growth factor I and B cell growth factor II, were produced by a variety of lymphocytes and non-lymphocytes, which can promote proliferation of B cells, enhance their ability to express and induce to produce IgG and IgA antibodies. IgA is a secretory immunoglobulin which expressed strongly in the respiratory, alimentary and reproductive tracts, and plays important roles in antibody-mediated defenses against a variety of viruses. IgG is the most abundant immunoglobulin in serum (75%∼80% of the total) and it is also the main antibody that induced humoral immunity (Yun et al., 2000). In this study, IL-6 level of ducks in experimental groups were slight lower than those of the control group ducks at first 9 dpi, which is probably because of immunosuppression caused by FAdV-4 infection (Schonewille et al., 2008). The levels of IL-4 and IL-6 in intranasal injection group significantly increased from 9 dpi, being consistent with the up-regulation of IgA and IgG. IgM is the earliest immunoglobulin molecule produced by B cells and it is earliest antibody expressed on the surface of B cells. Following FAdV-4 infection, infected ducks showed high levels of IgM at first 9 dpi, which indicated that IgM was significant in early pathogen infection.

In summary, although most of experimental muscovy ducks had no major clinical symptoms and no death, gross and histopathological lesions were obvious. In addition, virus replication was induced a strong immune response. This study indicated changes of antibodies levels, cytokines levels and biochemical indices in serum caused by HPS and provided the basis for future investigations.

## Author Contributions

YXD, YT, XY, and ZW conceived and designed the experiments. XY, HC, and YGD performed the experiments. XY, JY, and XN analyzed the data. XY and ZW contributed reagents, materials, and analysis tools. XY wrote the paper.

## Conflict of Interest Statement

The authors declare that the research was conducted in the absence of any commercial or financial relationships that could be construed as a potential conflict of interest. The reviewer TS and the handling Editor declared their shared affiliation.
